# Genome sequence of *Phaeobacter inhibens* type strain (T5^T^), a secondary metabolite producing representative of the marine *Roseobacter* clade, and emendation of the species description of *Phaeobacter inhibens*

**DOI:** 10.4056/sigs.4448212

**Published:** 2013-12-15

**Authors:** Marco Dogs, Sonja Voget, Hazuki Teshima, Jörn Petersen, Karen Davenport, Hajnalka Dalingault, Amy Chen, Amrita Pati, Natalia Ivanova, Lynne A. Goodwin, Patrick Chain, John C. Detter, Sonja Standfest, Manfred Rohde, Sabine Gronow, Nikos C. Kyrpides, Tanja Woyke, Meinhard Simon, Hans-Peter Klenk, Markus Göker, Thorsten Brinkhoff

**Affiliations:** 1 Institute for Chemistry and Biology of the Marine Environment (ICBM), Oldenburg, Germany; 2 Department of Genomic and Applied Microbiology and Göttingen Genomics Laboratory, Institute of Microbiology and Genetics, University of Göttingen, Göttingen, Germany; 3 Los Alamos National Laboratory, Bioscience Division, Los Alamos, New Mexico, USA; 4 Leibniz Institute DSMZ - German Collection of Microorganisms and Cell Cultures, Braunschweig, Germany; 5 Biological Data Management and Technology Center, Lawrence Berkeley National Laboratory, Berkeley, California, USA; 6 DOE Joint Genome Institute, Walnut Creek, California, USA; 7 HZI – Helmholtz Centre for Infection Research, Braunschweig, Germany

**Keywords:** Anaerobic, motile, rod-shaped, tropodithietic acid, secondary metabolites, *Rhodobacterales*, *Rhodobacteraceae*

## Abstract

Strain T5^T^ is the type strain of the species *Phaeobacter inhibens* Martens *et al.* 2006, a secondary metabolite producing bacterium affiliated to the *Roseobacter* clade. Strain T5^T^ was isolated from a water sample taken at the German Wadden Sea, southern North Sea. Here we describe the complete genome sequence and annotation of this bacterium with a special focus on the secondary metabolism and compare it with the genomes of the *Phaeobacter inhibens* strains DSM 17395 and DSM 24588 (2.10), selected because of the close phylogenetic relationship based on the 16S rRNA gene sequences of these three strains. The genome of strain T5^T^ comprises 4,130,897 bp with 3.923 protein-coding genes and shows high similarities in genetic and genomic characteristics compared to *P. inhibens* DSM 17395 and DSM 24588 (2.10). Besides the chromosome, strain T5^T^ possesses four plasmids, three of which show a high similarity to the plasmids of the strains DSM 17395 and DSM 24588 (2.10). Analysis of the fourth plasmid suggested horizontal gene transfer. Most of the genes on this plasmid are not present in the strains DSM 17395 and DSM 24588 (2.10) including a nitrous oxide reductase, which allows strain T5^T^ a facultative anaerobic lifestyle. The G+C content was calculated from the genome sequence and differs significantly from the previously published value, thus warranting an emendation of the species description.

## Introduction

Strain T5^T^ was isolated from a water sample taken on 25^th^ of October 1999 above an intertidal mud flat of the German Wadden Sea (53°42’20’’N, 07°43’11’’E) and found to be closely related to the type strain of *Roseobacter gallaeciensis* [1]. Two years later Martens *et al.* (2006) reclassified *Roseobacter gallaeciensis* as *Phaeobacter gallaeciensis* and described strain T5^T^ as type strain of the species *Phaeobacter inhibens*. As found for various *Phaeobacter* strains [2-7], *P. inhibens* strain T5^T^ (= DSM 16374^T^ = LMG 22475^T^ = CIP 109289^T^) is able to produce the antibiotic tropodithietic acid (TDA) [8]. Furthermore, strains of *P. gallaeciensis* and *P. inhibens*, including strain T5^T^, are able to produce a brownish pigment, which is the basis of the genus name (*phaeos* = dark, brown) [1]. The epithet of the species name points to the strong inhibitory activity of *P. inhibens* against different taxa of marine bacteria and algae [1]. The genus *Phaeobacter* is known to have a high potential for secondary metabolite production, as indicated by biosynthesis of TDA and *N-*acyl homoserine lactones (AHLs), as well as presence of genes coding for polyketide synthases (PKS) and nonribosomal peptide synthetases (NRPS) [2,7-10]. Biosynthesis of many different bioactive natural products is mediated by PKSs or NRPSs, including antibiotics, toxins and siderophores. Moreover, production of volatile compounds is widespread over the *Roseobacter* clade. It displays a particularly high proportion of volatile sulfur-containing compounds and thus seems to play an important role in the sulfur cycle of the ocean [11]. The sulfur-containing TDA, for which the sulfur precursor has not yet been determined, plays an important role in the mutualistic symbioses of *P. inhibens* and marine algae [12]. *p*-Coumaric acid causes the organism to switch from a state of mutualistic symbiosis to a pathogenic lifestyle in which toxicity is mediated via the production of the algicidal roseobacticides, which, like p-coumaric, is also a sulfur-containing metabolite [13,14].

Here we present the genome of *P. inhibens* strain T5^T^ with particular emphasis on the genes involved in secondary metabolism and comparison with the recently published genomes of the *P. inhibens* strains DSM 17395 and DSM 24588 (2.10) [3]. DSM 17395 and DSM 24588, originally deposited as *P. gallaeciensis* strains, were recently reclassified as *P. inhibens* [15].

## Classification and features

### 16S rRNA gene analysis

**Figure 1:**
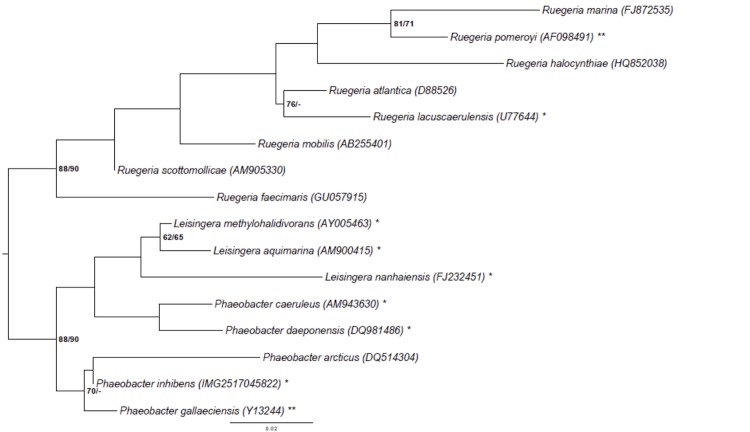
Phylogenetic tree highlighting the position of *P. inhibens* relative to the type strains of the other species within the genus *Phaeobacter* and the neighboring genera *Leisingera* and *Ruegeria* [1,20-33]. The tree was inferred from 1,385 aligned characters [34,35] of the 16S rRNA gene sequence under the maximum likelihood (ML) criterion [36]. Rooting was done initially using the midpoint method [37] and then checked for its agreement with the current classification (Table 1). The branches are scaled in terms of the expected number of substitutions per site. Numbers adjacent to the branches are support values from 1,000 ML bootstrap replicates [38] (left) and from 1,000 maximum-parsimony bootstrap replicates [39] (right) if larger than 60%. Lineages with type strain genome sequencing projects registered in GOLD [40] are labeled with one asterisk, those also listed as 'Complete and Published' with two asterisks [21]. The genomes of six more *Leisingera* and *Phaeobacter* species are published in the current issue of *Standard in Genomic Science* [41-46]. The 16S rRNA sequences of *P. inhibens* strain DSM 24588 and *P. inhibens* strain DSM 17395 are virtually identical to those of *P. inhibens* DSM 16374^T^ (data not shown).

Figure 1 shows the phylogenetic neighborhood of *P. inhibens* DSM 16374^T^ in a tree based on 16S rRNA genes. The sequences of the three identical 16S rRNA gene copies differ by one nucleotide from the previously published 16S rRNA sequence (NCBI Accession No. AY177712).

**Table 1:** Classification and general features of *P. inhibens* T5^T^ according to the MIGS recommendations [48].

MIGS ID	Property	Term	Evidence code
	Current classification	Domain *Bacteria*	TAS [49]
		Phylum *Proteobacteria*	TAS [50]
		Class *Alphaproteobacteria*	TAS [51,52]
		Order *Rhodobacterales*	TAS [52]
		Family *Rhodobacteraceae*	TAS [52,53]
		Genus *Phaeobacter*	TAS [1]
		Species *Phaeobacter inhibens*	TAS [1]
		Type strain T5	TAS [1,8]
	Gram stain	negative	TAS [1]
	Cell shape	rod shaped	TAS [1]
	Motility	motile	TAS [1]
	Sporulation	none	TAS [1]
MIGS-6.1	Temperature range	mesophile	TAS [1]
MIGS-6.1	Optimum temperature	27-29°C	TAS [1]
MIGS-6.3	Salinity	0.01- <1.5M NaCl	TAS [1]
MIGS-22	Oxygen requirement	facultative anaerobic	IDA
	Carbon source	oligosaccharides, sugar alcohols, organic acids, amino acids	TAS [1]
	Energy metabolism	heterotrophic	TAS [1]
MIGS-6	Habitat	marine	TAS [1]
MIGS-15	Biotic relationship	unknown	NAS
MIGS-14	Pathogenicity	none	TAS [1]
	Biosafety level	1	TAS [54]
MIGS-23	Isolation	water sample above an intertidal mud flat	TAS [1,8]
MIGS-4	Geographic location	German Wadden Sea	TAS [1,8]
MIGS-5	Sample collection time	October 25, 1999	TAS [1,8]
MIGS-4.1	Latitude	53°42´20´´N	TAS [1,8]
MIGS-4.2	Longitude	07°43´11´´E	TAS [1,8]
MIGS-4.3	Depth	Above sea ground	TAS [1,8]
MIGS-4.4	Altitude	unknown	

Evidence codes – IDA: Inferred from direct assay; TAS: Traceable Author Statement (i.e., a direct report exists in the literature); NAS: Non-traceable Author Statement (i.e., not directly observed for the living, isolated sample, but based on a generally accepted property for the species, or anecdotal evidence). Evidence codes are from the Gene Ontology project [55].

A representative genomic 16S rRNA gene sequence of *P. inhibens* DSM 16374^T^ was compared using NCBI BLAST [16,17] under default settings (e.g., considering only the high-scoring segment pairs (HSPs) from the best 250 hits) with the most recent release of the Greengenes database [18] and the relative frequencies of taxa and keywords (reduced to their stem [19]) were determined, weighted by BLAST scores. The most frequently occurring genera were *Ruegeria* (32.5%), *Phaeobacter* (28.8%), *Silicibacter* (13.6%), *Roseobacter* (13.3%) and *Nautella* (3.5%) (141 hits in total). Regarding the single hit to sequences from the species, the average identity within HSPs was 99.8%, whereas the average coverage by HSPs was 99.3%. Regarding the nine hits to sequences from other species of the genus, the average identity within HSPs was 99.0%, whereas the average coverage by HSPs was 99.2%. Among all other species, the one yielding the highest score was *P. gallaeciensis* (NZ_ABIF01000004), which corresponded to an identity of 100.0% and an HSP coverage of 100.0%. (Note that the Greengenes database uses the INSDC (= EMBL/NCBI/DDBJ) annotation, which is not an authoritative source for nomenclature or classification). The highest-scoring environmental sequence was AJ296158 (Greengenes short name 'Spain:Galicia isolate str. PP-154'), which showed an identity of 99.8% and an HSP coverage of 100.0%. The most frequently occurring keywords within the labels of all environmental samples which yielded hits were 'microbi' (3.1%), 'marine' (2.6%), 'coral' (2.3%), 'biofilm' (2.1%) and 'membrane, structure, swro' (1.8%) (100 hits in total). Environmental samples which yielded hits of a higher score than the highest scoring species were not found.

### Morphology and physiology

**Figure 2:**
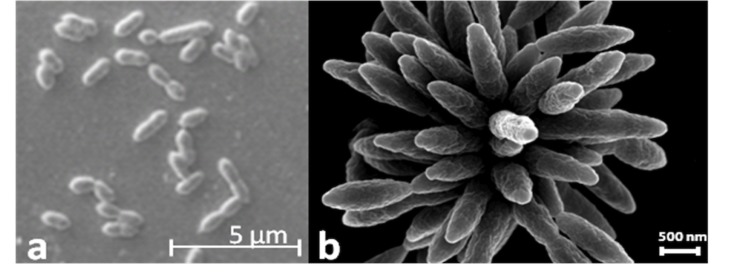
Scanning electron microscope pictures of *P. inhibens* strain DSM 16374^T^ showing (a) the typical cell ovoid shape of strain T5^T^ and (b) the multicellular, star-shaped structure as described previously for *Phaeobacter* and further *Roseobacter*-clade organisms.

Cells of T5^T^ are ovoid rods, 1.4-1.9 x 0.6-0.8 µm (Figure 2). Furthermore, T5^T^ cells show the typical multicellular star-shaped structure described previously for *P. gallaeciensis* and other *Roseobacter*-clade organisms [2,4,47] (Figure 2). Cells of T5^T^ are motile by means of a polar flagellum. T5^T^ is a Gram-negative, marine, facultatively anaerobic, mesophilic bacterium with an optimal growth temperature between 27 and 29 °C and an optimal salinity between 0.51 and 0.68 M. The pH range for growth is 6.0 – 9.5, with an optimum at 7.5. On marine agar T5^T^ forms smooth and convex colonies with regular edges and brownish pigmentation on ferric citrate containing media. T5^T^ utilizes pentoses, hexoses, disaccharides and most amino acids as carbon and energy sources. No vitamin requirements were observed [1].

### Chemotaxonomy

There are no significant differences between the fatty-acid profile of strain T5^T^ and other representatives of the *Roseobacter* clade [1]. Strain T5^T^ has the highest profile similarity to *P. gallaeciensis* CIP 105210^T^ [1]. The principal cellular fatty acids of strain T5^T^ are the following saturated branched-chain fatty acids: C_18:1ω7c_ (73.77%), 11-methyl C_18:1ω7c_ (7.45%), C_16:0_ (3.83%), C_18:0_ (3.14%), 2-OH C_16:0_ (3.10%), C_14:1_ (2.19%), 3-OH C_10:0_ (1.71%), 3-OH C_12:0_ (1.59%), 3-OH C_14:1_/ 3 oxo-C_14:0_ (0.87%), C_18.1ω9c_ (0.76%) and an unambiguously identified fatty acid (1.59%) [1]. The major polar lipids of strain T5^T^ comprise phosphatidylglycerol, phosphatidylethanolamine, phosphatidylcholine, an aminolipid and two unidentified lipids [1].

## Genome sequencing and annotation

### Genome project history

This organism was selected for sequencing on the basis of the DOE Joint Genome Institute Community Sequencing Program (CSP) 2010, CSP 441 “Whole genome type strain sequences of the genera *Phaeobacter* and *Leisingera* – a monophyletic group of physiological highly diverse organisms”. The genome project is deposited in the Genomes On Line Database [40] and the complete genome sequence is deposited in GenBank and the Integrated Microbial Genomes database (IMG) [56]. Sequencing, finishing and annotation were performed by the DOE Joint Genome Institute (JGI) using state of the art sequencing technology [57]. A summary of the project information is shown in Table 2.

**Table 2:** Genome sequencing project information

MIGS ID	Property	Term
MIGS-31	Finishing quality	Permanent draft
MIGS-28	Libraries used	Two Illumina paired-end libraries (225 bp and 9kb insert size)
MIGS-29	Sequencing platforms	Illumina GAii, PacBio
MIGS-31.2	Sequencing coverage	1,111 × Illumina
MIGS-30	Assemblers	Allpaths, Velvet 1.1.05, phrap version SPS - 4.24
MIGS-32	Gene calling method	Prodigal 1.4, GenePRIMP
	INSDC ID	AXBB00000000
	GenBank Date of Release	September 30, 2013
	GOLD ID	Gi10860
	NCBI project ID	88111
	Database: IMG-GEBA	2516653078
MIGS-13	Source material identifier	DSM 16374
	Project relevance	Tree of Life, carbon cycle, sulfur cycle, environmental

### Growth conditions and DNA isolation

A culture of DSM 16374^T^ was grown aerobically in DSMZ medium 514 [58] at 25°C. Genomic DNA was isolated using the Jetflex Genomic DNA Purification Kit (GENOMED 600100) following the standard protocol provided by the manufacturer but modified by an incubation time of 40 min, the incubation on ice over night on a shaker, the use of additional 10 µl proteinase K, and the addition of 100 µl protein precipitation buffer. DNA is available from DSMZ through the DNA Bank Network [59].

### Genome sequencing and assembly

For this genome, we constructed and sequenced an Illumina short-insert paired-end library with an average insert size of 225 bp, and an Illumina long-insert paired-end library with an average insert size of 9602 bp, which generated 18,471,132 reads and 11,906,846 reads, respectively, totaling 4,557 Mbp of Illumina data. All general aspects of library construction and sequencing performed can be found at the JGI website [60]. The initial draft assembly contained 13 contigs in 10 scaffold. The initial draft data was assembled with Allpaths [61] and the consensus was computationally shredded into 10 kbp overlapping fake reads (shreds). The Illumina draft data was also assembled with Velvet [62], and the consensus sequences were computationally shredded into 1.5 kbp overlapping fake reads (shreds). The Illumina draft data was assembled again with Velvet using the shreds from the first Velvet assembly to guide the next assembly. The consensus from the second Velvet assembly was shredded into 1.5 kbp overlapping fake reads. The fake reads from the Allpaths assembly and both Velvet assemblies and a subset of the Illumina CLIP paired-end reads were assembled using parallel phrap (High Performance Software, LLC) [63]. Possible mis-assemblies were corrected with manual editing in Consed [63]. Gap closure was accomplished using repeat resolution software (Wei Gu, unpublished), and sequencing of bridging PCR fragments with PacBio technologies. A total of 10 PCR PacBio consensus sequences were completed to close gaps and to raise the quality of the final sequence. The final assembly is based on 4,557 Mbp of Illumina draft data, which provides an average 1,111 × coverage of the genome.

### Genome annotation

Genes were identified using Prodigal [64] as part of the DOE-JGI genome annotation pipeline [65], followed by a round of manual curation using the JGI GenePRIMP pipeline [66]. The predicted CDSs were translated and used to search the National Center for Biotechnology Information (NCBI) nonredundant database, UniProt, TIGR-Fam, Pfam, PRIAM, KEGG, COG, and InterPro databases. Additional gene prediction analysis and functional annotation were performed within the Integrated Microbial Genomes - Expert Review (IMG-ER) platform [56].

## Genome properties

**Figure 3:**
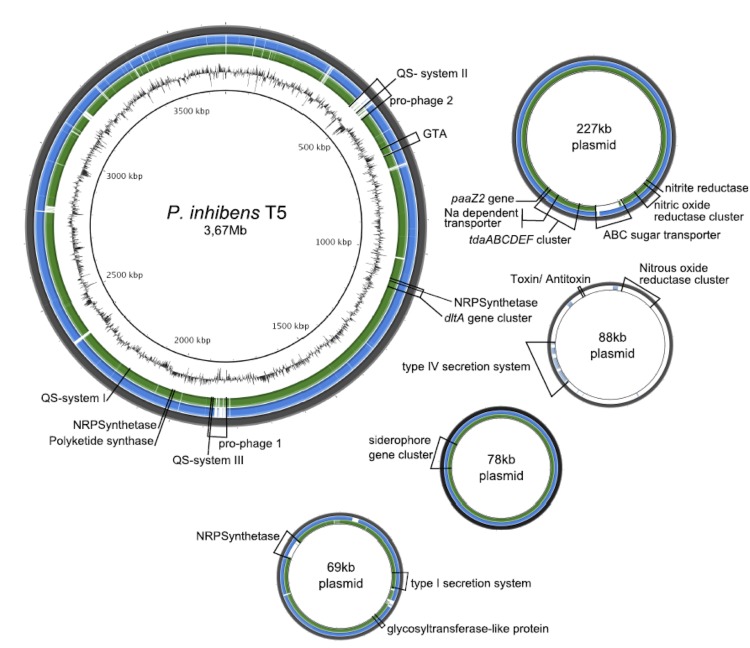
Graphical representation of the genome of *P. inhibens* T5^T^. From outside to the center: (1) sequence of *P. inhibens* T5^T^, (2) results of a blastn comparison from *P. inhibens* DSM 24588 (2.10) against *P. inhibens* T5^T^, (3) results of a blastn comparison of *P. inhibens* DSM 17395 against *P. inhibens* T5^T^, (4) G+C content. Comparisons and visualization are done with BRIG [67].

The genome statistics are provided in Table 3 and Figure 3. The genome consists of six scaffolds with a total length of 4,130,897 bp and a G+C content of 60.0%. The scaffolds correspond to a chromosome 3,669,861 bp in length and four extrachromosomal elements as identified by their replication systems (see below). Of the 3,986 genes predicted, 3,923 were protein-coding genes, and 63 RNAs; 39 pseudogenes were also identified. The majority of the protein-coding genes (81.0%) were assigned a putative function while the remaining ones were annotated as hypothetical proteins. The distribution of genes into COGs functional categories is presented in Table 4.

**Table 3:** Genome Statistics

**Attribute**	Value	% of total
Genome size (bp)	4.130.897	100.00%
DNA coding region (bp)	3.683.922	89.18%
DNA G+C content (bp)	2.479.086	60.02%
Number of scaffolds	6*	
Extrachromosomal elements	4	
Total genes	3.986	100.00%
RNA genes	63	1.58%
rRNA genes	5*	0.13%
rRNA operons	1*	
Protein-coding genes	3.923	98.42%
Pseudo genes	39	0.98%
Genes with function prediction	3.228	80.98%
Genes in paralog clusters	1.234	30.96%
Genes assigned to COGs	3.178	79.73%
Genes assigned Pfam domains	3.395	85.17%
Genes with signal peptides	306	7.68%
Genes with transmembrane helices	841	21.10%
CRISPR repeats	0	

* An updated version of the genome assembly (unpublished data) reveals the presence of four rRNA operons. In this version, two of the rRNA operons are incomplete, with the 16S rRNA gene and the 5S rRNA gene hidden in the two remaining sequencing gaps. The genomes of *P. inhibens* strain DSM 24588 (2.10) and *P. inhibens* strain DSM 17395 (see Figure 3) also contain four rRNA operons.

**Table 4:** Number of genes associated with the general COG functional categories

Code	Value	%age	Description
J	170	4.87	Translation, ribosomal structure and biogenesis
A	1	0.03	RNA processing and modification
K	274	7.85	Transcription
L	137	3.92	Replication, recombination and repair
B	3	0.09	Chromatin structure and dynamics
D	29	0.83	Cell cycle control, cell division, chromosome partitioning
Y	n. a.	n. a.	Nuclear structure
V	45	1.29	Defense mechanisms
T	156	4.47	Signal transduction mechanisms
M	199	5.70	Cell wall/membrane biogenesis
N	52	1.49	Cell motility
Z	n. a.	n. a.	Cytoskeleton
W	n. a.	n. a.	Extracellular structures
U	60	1.72	Intracellular trafficking and secretion, and vesicular transport
O	127	3.64	Posttranslational modification, protein turnover, chaperones
C	204	5.84	Energy production and conversion
G	197	5.64	Carbohydrate transport and metabolism
E	425	12.17	Amino acid transport and metabolism
F	79	2.26	Nucleotide transport and metabolism
H	152	4.35	Coenzyme transport and metabolism
I	146	4.18	Lipid transport and metabolism
P	174	4.98	Inorganic ion transport and metabolism
Q	110	3.15	Secondary metabolites biosynthesis, transport and catabolism
R	446	12.77	General function prediction only
S	306	8.76	Function unknown
-	808	20.27	Not in COGs

## Insights into the genome

Genome sequencing of *P. inhibens* DSM 16374^T^ revealed the presence of four extrachromosomal elements with sizes of 227 kb, 88 kb, 78 kb, and 69 kb (Figure 3; Table 5) and DnaA-like I, RepABC-8, RepB-I and RepA-I as replication systems, respectively [68]. The different replicases that mediate the initiation of replication are designated according to the established plasmid classification scheme [69]. With the exception of the 88 kb replicon, these extrachromosomal elements are highly syntenic to specific replicons in the genomes of *P. inhibens* strains DSM 17395 and DSM 24588 (Figure 3).

**Table 5:** General genomic features of the chromosome and extrachromosomal replicons of *Phaeobacter inhibens* strain DSM 16374^T^

**Replicon**	**Scaffold**	**Length** (bp)	**GC** (%)	**Topology**	**No. Genes^#^**
cInhi_A3361	1	3,361,358	60	linear*	3,282
cInhi_B309	2	308,503	61	linear*	288
pInhi_A227	3	226,687	59	linear*	208
pInhi_B88	4	87,579	58	linear*	93
pInhi_C78	5	78,203	63	linear*	62
pInhi_D69	6	68,567	63	linear*	53

^#^deduced from automatic annotation

*circularity not experimentally validated.

The locus tags of all replicases, plasmid stability modules and the large *virB4* gene of a type IV secretion system are presented in Table 6. The plasmids pInhi_A227 and pInhi_B88 contain postsegregational killing systems (PSK) consisting of a typical operon with two small genes encoding a stable toxin and an unstable antitoxin [70]. Moreover, plasmid pInhi_B88 also contains a complete *virB* gene cluster of type IV secretion system, required for the formation of a transmembrane channel. However, the absence of the relaxase VirD2, which is necessary for the strand-specific DNA nicking at the origin of transfer (*oriT*), and the coupling protein VirD4 indicates that this plasmid is non-conjugative [71,72]. The RepA-I type replicon pInhi_D69 contains a complete rhamnose operon [73] and is dominated by genes required for polysaccharide biosynthesis.

**Table 6:** Integrated Microbial Genome (IMG) locus tags of *P. inhibens* DSM 16374^T^ genes for the initiation of replication, toxin/antitoxin modules and two representatives of type IV secretion systems (T4SS) that are required for conjugation.

**Replicon**	**Replication Initiation**	**Plasmid Stability**	**Type IV Secretion**	**Replicon**	**Replication Initiation**	**Plasmid Stability**
	Replicase	Locus Tag	Toxin	Antitoxin	VirB4	VirD4
cInhi_A3361	DnaA	Inhi_2434	-	-	-	-
cInhi_B309	-	-	-	-	-	-
pInhi_A227	DnaA-like I	Inhi_3576	Inhi _3735	Inhi _3734	-	-
pInhi_B88	RepC-8	Inhi_3797	Inhi _3865	Inhi _3866	Inhi _3845	-
pInhi_C78	RepB-I	Inhi_3883	-	-	-	-
pInhi_D69	RepA-I	Inhi 3972	-	-	-	-

As already indicated by the strong inhibitory activity of *P. inhibens* T5^T^ [8] all 26 described genes involved in the production of TDA are present in the genome of this strain. As found for the *P. inhibens* strains DSM 17395 and DSM 24588, the key genes for TDA production *tdaABCDEF* (Inhi_3684 - _3688, Inhi_3701), *paaZ2* (Inhi_3702) and a gene coding for a putative Na-dependent transporter (Inhi_3697) [3,74] are located on the 227 kb plasmid of T5^T^ (Figure 3). The remaining 19 genes, containing genes of the phenylacetyl-CoA and assimilatory sulfate reduction pathways, are scattered over the chromosome as in the strains DSM 17395 and DSM 24588 [3]. Beside the *tdaA* gene, present on the 227 kb plasmid, we also found other genes involved in the regulation of TDA synthesis located on the chromosome, what is in agreement with Thole *et al.* (2012) and Berger *et al.* (2012) [3,75]. This includes the genes encoding transcriptional activator proteins (Inhi_2121; _2059; _0396) comparable with *pgaR*, *iorR* a transcriptional regulator (PGA1_c20730), a putative serine-protein kinase (Inhi_2265) and a putative signal peptide peptidase (Inhi_2227).

Two complete prophages and an additional cluster coding for the production of gene transfer agents (GTA) were found in the genome of strain T5^T^. The GTA gene cluster is equal in length and comprises the same genes (Inhi_0654 – Inhi_0670) as the GTA clusters of the strains DSM 17395 and DSM 24588. The two prophages of strain T5^T^ consist of 52 ORFs (prophage 1; ~37kb) and 63 ORFs (prophage 2; ~48kb), respectively. Strain DSM 17395 possesses two prophages, but for DSM 24588 no prophages were detected [3]. Prophage 1 of strain T5^T^ is similar to prophage 1 of strain DSM 17395, with the exception that a few ORFs are different (PGA1_c18280 - _c18310, PGA1_c18480 - _c18530 and PGA1_c18570 - _c18680; Inhi_1777, Inhi_1785 - _1788, Inhi_1803 - _1812 and Inhi_1816 – 1829). Prophage 2 of strain T5^T^ is a Mu-like bacteriophage, not present in strain DSM 17395.

It was previously shown that strain T5^T^ produces two different AHLs, i.e. C_18-en_-HSL and N-3-hydroxydecanoyl-homoserine lactone (3OHC_10_-HSL) [76]. In *P. inhibens* strain DSM 17395 TDA and pigment production are regulated via a *pgaR*-*pgaI* QS system [47]. The AHL synthase encoding gene *pgaI* in DSM 17395 is responsible for the production of 3OHC_10_-HSL. In the genome of strain T5^T^ we found a homologous system probably coding for the 3OHC_10_-HSL producing AHL synthase (Inhi_2120, homolog to *pgaI*) and the respective regulator (Inhi_2121, homologous to *pgaR*) (Figure 3, QS system I). Thus TDA production in strain T5^T^ might also be regulated by a QS system. In addition, two further QS systems (QS system II and III; Figure 3) were found on the chromosome of T5^T^. System II is formed by the genes Inhi_0506 and _0507 and is located in the prophage region 2. Orthologs for these QS system genes are also present in *P. inhibens* strain DSM 24588 (PGA2_c18960 and PGA2_c18970) but absent in strain DSM 17395. QS system III consists of the genes Inhi_1819 and _1820 and is unique for strain T5^T^ compared to *P. inhibens* DSM 17395 and DSM 24588. It is also located in the potential prophage 1 region (Fig. 3). A homologous system was found in the genome of *Phaeobacter caeruleus* DSM 24564^T^ and the neighboring genes show a high synteny. The location in the prophage region and the high synteny to the system of *P. caeruleus* suggest a possible gene transfer of this QS system via a bacteriophage. The functions of QS system II and III are currently unknown, but it is likely that the compound C_18_-en-HSL is produced by one of those systems.

Two functions were suggested that can possibly be used as unique chemotaxonomic markers for the species *P. inhibens* within the *Roseobacter* clade [3]. The genes coding for the first of these functions are located on the chromosome and are involved in cell wall development and surface attachment [*dltA* encoding a D-alanine-poly(phosphoribitol) ligase involved in biosynthesis of D-alanyl-lipoteichoic acid]. The second unique function is the biosynthesis and transport of iron-chelating siderophores, and the encoding genes are located on the plasmid pPGA1_78 and pPGA2_95, respectively. These two clusters are also present in the genome of strain T5^T^. The siderophore gene cluster (Inhi_3924 – Inhi_3928) is located on the 78 kb plasmid (Fig. 3) and the *dltA* gene cluster (Inhi_1065 - Inhi_1086) is located on the chromosome (Fig. 3). Screenings in the newly available *Roseobacter* genomes showed that *Leisingera methylohalidivorans* DSM 14336 [42] and *Leisingera aquimarina* DSM 24565 [41] also harbor the genes for siderophore synthesis. The uniqueness of the *dltA* gene cluster within the species *P. inhibens*, however, remains and can be used as chemotaxonomic marker.

The existence of genes coding for a polyketide synthase (Inhi_1972) and three non-ribosomal peptide synthetases (Inhi_1072, _1974 and _3983) confirm the results of Martens *et al.* (2007) [7]. These genes are present in the genomes of strains DSM 17395 and DSM 24588, too (PGA1_c04930 and PGA1_c05350, _c13760, _c28490; PGA2_c05370 and PGA2_c04910, _c13660, _71p110). The genes Inhi_3983 of *P. inhibens* strain T5^T^ and PGA2_71p110 of *P. inhibens* strain DSM 24588 are located on the 69 kb plasmid (Fig. 3) and 71 kb plasmid, respectively. In contrast, the homologous gene (PGA1_c28490) of *P. inhibens* strain DSM 17395 is located on the chromosome.

For the *P. inhibens* strains DSM 17395 and DSM 24588 a surface-attached lifestyle was inferred from the genome analysis [3]. Even though strain T5^T^ was isolated from a water sample, it exhibits the same genes associated with the biosynthesis and transport of polysaccharides as strains DSM 17395 and DSM 24588. This includes genes described as unique for the strains DSM 17395 and DSM 24588, i.e. a gene coding for a glycosyltransferase-like protein (Inhi_3961) and two ORFs (Inhi_3954 and Inhi_3955) related to a type I secretion system and used for the transport of exopolysaccharides. Production of extracellular polysaccharides is a major factor contributing to surface attachment [77,78]. Thus it appears likely that T5^T^ is also well-adapted to a surface attached lifestyle.

*P. inhibens* was described as a strictly aerobic bacterium [1]. However, we found genes involved in the dissimilatory nitrate reduction pathway to nitrogen, including the gene coding for a copper containing nitrite reductase (Inhi_3645) and a nitric oxide reductase cluster (Inhi_3648 - Inhi_3654), both located on the replicon pInhi_A227. These genes are also present and located on the largest plasmids of *P. inhibens* DSM 17395 (PGA1_262p) and *P. inhibens* DSM 24588 (PGA2_239p) (Figure 3). In addition, *P. inhibens* strain T5^T^ possesses a gene cluster coding for a nitrous oxide reductase (Inhi_3786 – Inhi_3792) located on the replicon pInhi_B88, which is absent in the strains DSM 17395 and DSM 24588 (Figure 3). Neither strain T5^T^ nor DSM 17395 and DSM 24588 have genes coding for a nitrate reductase. The findings suggest that *P. inhibens* T5^T^ has a complete dissimilatory nitrite reduction pathway, but is not able to reduce nitrate, as previously described by Martens *et al.* (2006) [1]. To confirm the results we tested strain T5^T^ for its capability to grow anaerobically with nitrite. Anaerobic marine basal medium was prepared according to Cypionka and Pfennig (1986) [79] and supplemented with nitrite and glucose, both in a final concentration of 5 mM. After two weeks a decrease of nitrite was determined by photometric analysis at 545 nm by using the Griess reaction [80] and an increase of the turbidity was detected (results not shown). Thus it became clear that *P. inhibens* T5^T^ is able to grow anaerobically with nitrite, suggesting an emended description of this organism as a facultatively anaerobic bacterium.

Phylogenetic analysis shows that *P. inhibens* and *P. gallaeciensis* form a cluster together with *Phaeobacter arcticus* (Figure 1). The cluster is set apart from the cluster comprising *Leisingera aquimarina*, *Leisnigera nanhaiensis*, *Leisingera methylohalidivorans*, *Phaeobacter caeruleus* and *Phaeobacter daeponensis*, but the backbone of the 16S rRNA gene tree shown in Figure 1 is rather unresolved. Using the online analysis tool “Genome-to-Genome Distance Calculator^”^ 2.0 (GGDC) [81,82], we performed a preliminary phylogenetic analysis of the draft genomes of the type strains of the genera *Leisingera* and *Phaeobacter* and the finished genomes of *P. inhibens* strains DSM 17395 and DSM 24588. Table 7 shows the results of the *in silico* calculated DNA-DNA hybridization (DDH) similarities of *P. inhibens* to other *Phaeobacter* and *Leisingera* species. In the following analysis, we will refer only to the results of formula 2, as this formula is robust against the use of draft genomes such as AOQA01000000 (CIP 105210^T^) [83]. The use of GGDC revealed a high similarity of T5^T^ (78%) to the strains *P. inhibens* DSM 17395 and DSM 24588, but a low similarity to *P. gallaeciensis* strain CIP 105210^T^ (36%). DSM 17395 and CIP 105210^T^ were previously supposed to be type-strain deposits for *P. gallaeciensis* [33] and we cross-compared them using GGDC. Formula 2 yielded a similarity of only 38.30% ± 2.50 between these two strains, thus indicating not only that they are not the same strain, but also do not even belong to the same species. The results are in agreement with the study of Buddruhs *et al.* (2013) [15] showing that strain DSM 17395 is the false deposit and belongs together with DSM 24588 to *P. inhibens*, whereas CIP 105210^T^ is the correct type-strain deposit for *P. gallaeciensis*.

**Table 7:** Digital DDH similarities between *P. inhibens* T5^T^ and the other *Phaeobacter* and *Leisingera* species (including the genome-sequenced type strains and *P. inhibens* strains DSM 17395 and DSM 24588 [2,10]) calculated *in silico* with the GGDC server version 2.0 [83]^†^.

**Reference strain (type strain unless indicated)**	**formula 1**	**formula 2**	**formula 3**
*P. arcticus* DSM 23566^T^ (AXBF00000000)	17.60±3.30	21.70±2.44	17.80±2.95
*P. caeruleus* DSM 24564^T^ (AXBI00000000)	19.00±3.36	20.60±2.46	19.10±2.99
*P. daeponensis* DSM 23529^T^ (AXBD00000000)	20.40±3.41	22.60±2.46	20.20±3.03
*P. gallaeciensis* CIP 105210^T^ (AOQA01000000)	78.40±3.76	36.20±2.57	68.50±3.52
*P. inhibens* DSM 17395 (CP002976, CP002977, CP002978, CP002979)	90.60±2.78	78.50±2.98	90.90±2.49
*P. inhibens* DSM 24588 (2.10) (CP002972, CP002973, CP002974, CP002975)	94.50±2.03	78.40±2.98	94.20±1.92
*L. aquimarina* DSM 24565^T^ (AXBE00000000)	18.50±3.34	22.50±2.45	18.70±2.98
*L. methylohalidivorans* DSM 14336^T^ (CP006773, CP006774, CP006775)	19.40±3.38	22.50±2.45	19.40±3.01
*L. nanhaiensis* DSM 24252^T^ (AXBG00000000)	14.00±3.08	21.00±2.42	14.70±2.78

^†^The standard deviations indicate the inherent uncertainty in estimating DDH values from intergenomic distances based on models derived from empirical test data sets (which are always limited in size); see [83] for details. The distance formulas are explained in [82]; formula 2 is recommended, particularly for draft genomes (such as AOQA01000000). The numbers in parentheses are GenBank accession numbers identifying the underlying genome sequences.

The differences in the G+C content (55.7%) published earlier [1] and the value calculated directly from the genome (Table 3) warrants an update of the taxonomic description on *P. inhibens* [84]. Moreover, genomic and experimental evidence indicates that *P. inhibens* is not strictly aerobic but facultatively anaerobic.

## Conclusion

### Emended description of the species *Phaeobacter inhibens* Martens *et al.* 2006

The description of the species *Phaeobacter inhibens* is the one given by Martens *et al.* 2006 [1], with the following modification. The G+C content, rounded to zero decimal places, is 60%. *Phaeobacter inhibens* is a facultative anaerobic bacterium by using nitrite reduction.
